# The Role of Exposure to Neighborhood and School Poverty in Understanding Educational Attainment

**DOI:** 10.1007/s10964-021-01427-x

**Published:** 2021-04-07

**Authors:** Jaap Nieuwenhuis, Tom Kleinepier, Maarten van Ham

**Affiliations:** 1 Department of Sociology, Zhejiang University, 866 Yuhangtang Road, Hangzhou, China; 2 ABF Research, Verwersdijk 8, Delft, the Netherlands; 3 Delft University of Technology & University of St. Andrews, Julianalaan 134, Delft, the Netherlands

**Keywords:** Neighborhoods, Schools, Poverty, Educational attainment, ALSPAC

## Abstract

Because the demographic composition of neighborhoods and schools overlaps, their effects on educational attainment are not independent of each other. Throughout the early teenage years, the timing and duration of exposure to neighborhood and school contexts can vary, advocating for a longitudinal approach when studying schooling outcomes. This study uses Avon Longitudinal Study of Parents and Children data (N = 4502; 49% female) to examine how exposure to poverty between ages 10–16 predicts educational attainment. The results indicate that enduring exposure to neighborhood poverty relates to educational attainment, while timing does not. For school poverty, longer exposure is related to lower attainment, but earlier exposure has a stronger impact than later exposure. Adolescents who were exposed to poverty in both contexts for the full observation period had the lowest educational attainment. The findings highlight the importance of understanding when and how long adolescents are exposed to contextual poverty.

## Introduction

With a few notable exceptions (see below), the field of contextual effects on educational outcomes rarely explicitly studies the neighborhood and school contexts simultaneously (Nieuwenhuis & Hooimeijer, 2016). There are many reasons to suspect that the effects of neighborhood and school poverty may overlap, intersect, or even reinforce each other. Schools are often located within or near neighborhoods where people live and where people have the same information about educational opportunities, making it likely that the demographic composition of both contexts will overlap to some extent. However, each might also have its own independent effect on individual outcomes. To measure contextual effects, a cross-sectional approach is often not sufficient. The duration (e.g., Nieuwenhuis et al., 2016) and the timing of exposure to a poor context matter (e.g., Alvarado, 2016). First, longer exposure to poverty is likely to have a stronger impact on outcomes than short-term exposure. Second, exposure to poverty at different ages will likely have a differential impact on adolescents’ outcomes. Here, a temporal perspective is used to examine the effects of neighborhood poverty and school poverty on adolescents’ educational attainment in the United Kingdom, by employing cross-classified multilevel models, to account for the clustering of individuals in both contexts. By studying adolescents at three ages (10/11, 13/14, and 15/16), the duration and timing of exposure to contextual poverty are taken into account. Using this approach, this study examines the importance of both neighborhood and school poverty at different ages in adolescents’ lives.

### Neighborhood Effects and/or School Effects

Both neighborhoods and schools are considered to exert an influence on adolescents’ educational development, however, because they cannot be seen independently from each other, it is important to study them simultaneously. Neighborhood effects researchers often argue for a relation between neighborhood poverty and educational outcomes, and they have proposed several mechanisms for it: For example, the presence of adult role models, supervision and social control may differ between neighborhoods; neighborhood-based social networks may provide different sources of information and resources pertaining to educational opportunities; and exposure to deviant peers can depend on the quality of the neighborhood (for an overview, see Galster, 2012). All these mechanisms address the demographic composition of neighborhoods and predict that the behavior of other residents influences the education-related behavior of local adolescents. However, it is very likely that the demographic composition of the neighborhood is also, to some extent, represented in the school. School catchment areas determine which schools parents can choose from, and these catchment areas are shared with neighbors, meaning that neighborhood children will end up within the same limited set of schools in the area. Poor neighborhoods are often faced with poor schools that have challenges attracting good teaching staff because of a lack of resources and a bad reputation (Wacquant, 2008). In catchment areas where schools are of low quality, parents may want to overcome this problem by moving or sending their children to private schools; however, especially in poorer neighborhoods, most parents will lack the resources to afford a better neighborhood or private school tuitions (Nieuwenhuis & Xu, 2021). In addition to catchment areas, geographical proximity in general affects the choice of institutions, because traveling time should remain manageable (Burgess et al., 2011). Because of the strong link between poverty and the demographic composition of neighborhoods and schools, studying them in tandem as among the multiple related groups to which adolescents belong is warranted (Nieuwenhuis & Hooimeijer, 2016).

Despite the relevance of school context for neighborhood effects studies, only approximately one-quarter of studies that examine the relation between neighborhood poverty and educational outcomes take into account school-related control variables (Nieuwenhuis & Hooimeijer, 2016). Those studies that do take these into account find a much weaker neighborhood effect than those studies that do not (Nieuwenhuis & Hooimeijer, 2016). The studies that specifically study the combined effects of neighborhoods and school on educational outcomes find mixed results. One study finds that the neighborhood effect disappears altogether after modeling the neighborhood and school together (Sykes & Musterd, 2011), while another finds that it remains (Bowen & Bowen, 1999). Others find partial mediation of the neighborhood effect through school factors (Ainsworth, 2002), no mediation by school poverty (Wodtke & Parbst 2017), that the results depend on how the neighborhood and school characteristics are measured (Owens, 2010; Pong & Hao, 2007), and that the neighborhood-level variance decreases after taking schools into account (Brännström, 2008; Kauppinen, 2008). Although the relation between neighborhood poverty and school poverty is not evident, most of the abovementioned studies do seem to suggest that they are not independent of each other.

### Duration of Exposure

The neighborhoods in which people live and the schools they attend are often not fixed. People move and transfer, leading to divergent neighborhood and school histories between people over their lifetimes. Some adolescents may spend their whole childhood in a disadvantaged neighborhood, while others may only live in one shortly (Kleinepier et al., 2018). Many of the mechanisms through which neighborhoods and schools are hypothesized to impact educational attainment are based on community characteristics (see Galster, 2012). Poor neighborhoods and schools have few social resources and poorer social organisation, and so children in such deprived contexts will be less stimulated to perform well in school. However, if adolescents are exposed to a deprived context for only a brief period, the impact it may have is arguably smaller compared to being exposed to poverty for the duration of childhood. Moreover, exposure to nondeprived contexts may even buffer the negative impact of the deprived context, because adolescents and their parents may retain resources and social networks from earlier nondeprived contexts. This notion is important because many studies of contextual poverty adopt a point-in-time measure of poverty rather than taking a longitudinal perspective (Nieuwenhuis & Hooimeijer, 2016). This has the disadvantage of only one certain moment in the life of adolescents being studied, while two adolescents who live in a poor neighborhood at the time of measurement may have had a very different unmeasured prior neighborhood history. Several studies have already found that a longitudinal approach to contextual poverty has more predictive power than a cross-sectional approach, suggesting that long-term exposure of up to six (Hicks et al., 2018) and up to 15 years in poverty (Wodtke et al., 2011) has greater impact on educational outcomes than short-term exposure.

### Timing of Exposure

In contrast to the increased attention given to the duration of exposure to neighborhood/school poverty in recent years, the developmental timing of exposure has received much less attention to date. Previous studies that take the timing of exposure into account have typically made a twofold distinction between early/middle childhood (ages 0–10 years) and adolescence (ages 11–20 years). These studies have consistently reported that exposure to poor neighborhoods during adolescence has stronger effects on individual outcomes than exposure to neighborhood poverty in early and/or middle childhood (e.g., Kleinepier & van Ham, 2018; Wodtke et al., 2016). An explanation for these findings has been sought in other studies showing that adolescents are more susceptible to peer pressure than are children or (young) adults (Prinstein & Dodge, 2008). Indeed, during adolescence, there is a sharp increase in the amount of time spent with peers (Brown, 2004). Moreover, this increase coincides with heightened sensation-seeking, risky behavior, and a growing contrast between peer and family values, for example, regarding the importance of doing well in school (Gardner & Steinberg, 2005). It was shown that more recent exposure to neighborhood disadvantage impacts educational outcomes more than less recent exposure (Hicks et al., 2018) and that removing children from poor neighborhoods before the age of 13 is the most beneficial (Chetty et al., 2015). This suggests that the studied educational outcomes of adolescents are most strongly affected by neighborhood/school resources, adult role models, and peer groups from the recent past.

### Cumulative Contextual Poverty

Growing up in a poor neighborhood and attending a poor school may have separate negative impacts on adolescents’ educational attainment. However, being exposed to poverty in both contexts effectively increases the overall exposure to poverty. Exposure to poverty in multiple contexts potentially has a larger impact on educational attainment than experiencing poverty in only one context (Mijs & Nieuwenhuis, 2018; Whipple et al., 2010). Being exposed to, for example, three periods of both school and neighborhood poverty is likely to have a very different impact on educational attainment than being exposed to three periods of school poverty, while simultaneously growing up in a middle-class or wealthy neighborhood.

### Selection

People do not end up in poor neighborhoods or schools by chance but select into these contexts (Galster, 2008). When ignoring how individuals select into places, spurious effects could arise that should actually be attributed to a characteristic that causes both the selection into the context as well as educational attainment. For adolescents, family socioeconomic background is such a characteristic, and it thus needs to be accounted for. Furthermore, by including a measure for educational attainment before the period in which the neighborhood and school poverty are assessed, part of the variance in educational attainment that is due to family background characteristics can be removed. Finally, when people move between neighborhoods, the move itself, or a change in situation, may cause spurious effects as well. Taking into account moving behavior can account for this.

## The Current Study

There is little understanding of how neighborhood and school poverty interact when studying educational outcomes because most studies look at only one context. Moreover, there is a lack of understanding of how the timing and duration of exposure to poverty in the two contexts combined impacts educational outcomes. This study aims to examine the relevance of school poverty when studying the effect of neighborhood poverty on educational attainment using data from a study from southwest England, United Kingdom. First, the relative importance of both neighborhood and school contexts is studied by using cross-classified multilevel models. Such models can account for the clustering of individuals within different non-nested contexts, in this case neighborhoods and schools. Selection is taken into account through family background, prior educational attainment, and moving behavior. Neighborhood poverty is defined comprehensively by including, among others, income, work, and education, as is frequently done (Nieuwenhuis & Hooimeijer, 2016). School poverty is defined by school meal eligibility, a common proxy in English education studies (Gorard, 2012). The importance of both contexts for educational attainment in general is examined by studying a comprehensive measure of educational attainment that includes scores from the national Key Stage 4 tests of reading, math, and science (taken at age 15/16). Key Stage 4 is the period during which students work towards national qualifications. The accompanying hypothesis is that the effect of neighborhood poverty on educational attainment will be smaller when taking into account school poverty (hypothesis 1). Second, the aim is to assess the importance of time within this neighborhood-school framework by specifically modeling the duration and timing of exposure to neighborhood and school poverty. Neighborhood and school poverty are measured at three different ages in the adolescents’ lives (i.e., at 10/11, 13/14, and 15/16). This allows for an understanding of differences between short-term and long-term exposure. The hypothesis is as follows: The longer adolescents are exposed to (a) neighborhood and (b) school poverty, the lower their educational attainment (hypotheses 2a and 2b). The temporal perspective also allows for a comparison of the differences between exposure at early ages and that at later ages in adolescence. Conforming to the literature review, the hypothesis is as follows: The relation between exposure to (a) neighborhood and (b) school poverty and educational attainment is stronger during later adolescence (ages 13–16 years) than during earlier adolescence (ages 10–11 years) (hypotheses 3a and 3b, respectively). Third, building on the previous points, neighborhood and school poverty are combined to study more general cumulative poverty during adolescence, leading to hypothesis 4: The longer adolescents are exposed to combined neighborhood and school poverty, the lower their educational attainment. With this cumulative measure the importance of the duration and timing of exposure to poverty is studied again, leading to hypothesis 5: The relation between exposure to combined neighborhood and school poverty and educational attainment is stronger during later adolescence (ages 13–16 years) than during earlier adolescence (ages 10–11 years).

## Data and Methods

### Participants

The Avon Longitudinal Study of Parents and Children (ALSPAC) is used to test the hypotheses. ALSPAC is an ongoing population based cohort study that recruited 14,541 pregnant women living in the county of Avon, UK, and who were expected to give birth between April 1st, 1991 and December 31st, 1992. The area in southwestern England contained approximately 900,000 inhabitants in 1991 and includes the city of Bristol and its surrounding areas. There was an additional enrollment of 713 children. The total sample consisted of 15,458 fetuses, of which 14,701 were alive at age 1 (Boyd et al., 2013; Fraser et al., 2013). Educational results from two national standardized tests taken at ages 7 and 15/16 and aggregated neighborhood and school information were obtained by linking ALSPAC data on children’s school and neighborhood histories to national databases, such as the Annual School Census and the National Pupil Database. The study sample consisted of 4502 adolescents who were clustered in 177 schools (on average, 25.4 respondents per school) and in 593 neighborhoods (on average, 7.6 respondents per neighborhood). Neighborhoods were defined as lower layer super output areas (LSOAs), which is the smallest available delineation (between 400 and 1200 households) (Office for National Statistics, 2020). Small delineations best capture the local social networks people are part of and affected by and are also most comparable to the scale of the experienced school environment (Nieuwenhuis et al., 2015). The sample was 49.3% female and aged 10/11 at the first used measurement point, 13/14 at the second, and 15/16 at the last.

The mothers in the data were more likely to be owner occupiers (79.1%) and car owners (90.8%) than the Avon average (68.7 and 83.7%) or the Great Britain average (63.4 and 75.6%). They were more likely to live in overcrowded housing (on average more than one person per room; 33.5%) than the Avon (26.0%) and Great Britain (30.8) averages. Participating mothers were more likely to be married (79.4%) than the populations of Avon (71.7%) and Great Britain (71.8%). Finally, non-White mothers were underrepresented in the data (2.2%) compared to Avon (4.1%) and Great Britain (7.6%). Attrition for follow-ups was higher among younger women and for women of a lower social class or with less education (Fraser et al., 2013). Similarly, children in the ALSPAC sample are more likely to have higher educational attainment and be White than the national average (Boyd et al., 2013). The data are a decent representation of the population, although the underrepresentation of lower-class people, less educated people, and people with a migrant background means that the results should be interpreted with caution for these groups. In the analyses, only adolescents with complete information on all variables were included, while others were listwise deleted. Several t-tests were run to examine whether the probability of missingness was associated with educational attainment (Allison, 2002). The tests show that only gender is missing at random (t = 1.0730, df = 14852, p = 0.2833). Not missing at random were duration of exposure to neighborhood poverty (t = −3.2227, df = 14192, p = 0.0013), duration of exposure to school poverty (t = −3.2072, df = 12621, p = 0.0013), prior educational achievement (t = 9.8242, df = 11557, p < 0.0001), parental education (t = −9.7484, df = 12519, p < 0.0001), and whether people moved (t = −46.8650, df = 14240, p < 0.0001). This selective attrition should be kept in mind when interpreting the results. Please note that the study website contains details of all the data available through a fully searchable data dictionary at http://www.bris.ac.uk/alspac/researchers/data-access/data-dictionary/.

### Variables

#### Educational attainment

The dependent variable is a factor score of three standardized test results (reading (range: 0–60), math (range: 0–68), and science (range: 0–58)) taken for the national Key Stage 4 test at age 15/16. These results were linked through the National Pupil Database (NPD). The factor analysis showed a clear indication of one factor, with factor loadings of 0.82 (reading), 0.88 (math), and 0.89 (science). Descriptive statistics and correlations for this and other variables can be found in Tables 1 and 2, respectively.

**Table 1:** Descriptive statistics (N = 4502)

Variable	Mean	SD	Min.	Max.
Educational attainment (factor score)	0.02	0.90	−3.70	1.92
Previous educational attainment	9.53	3.50	0	15
	%			
Parental education: CSE or GCSE (D–G)	14.68			
Vocational	7.26			
O level or GCSE (A–C)	30.61			
A level	32.92			
University degree	14.53			
Gender (female)	49.27			
Times moved: 0	87.32			
1	11.99			
2	0.69			
Moved between period 10/11 and 13/14	10.93			
Moved between period 13/14 and 15/16	2.44

**Table 2:** Correlations (N = 4502)

	1.	2.	3.	4.	5.	6.
1. Educational attainment	1					
2. Duration of exposure to neighborhood poverty	−0.2624*	1				
3. Duration of exposure to school poverty	−0.2354*	0.5319*	1			
4. Previous educational attainment	0.6768*	−0.1959*	−0.1636*	1		
5. Parental education	0.4254*	−0.2421*	−0.1800*	0.3109*	1	
6. Gender (female)	0.0703*	0.0102	−0.0025	0.1431*	0.0057	1
7. Moved	−0.0272	−0.0020	−0.0208	−0.0018	0.0012	0.0227

*p < 0.0001

#### Exposure to neighborhood poverty

Two contexts, the neighborhood and the school, at three time points, ages 10/11, 13/14, and 15/16, were used to measure individual exposure to contextual poverty. Neighborhood poverty was measured using government issued Indices of Multiple Deprivation (IMD) for the neighborhood (i.e., the LSOA) in which the adolescents lived during the three measurement periods (Payne & Abel, 2012). The IMD consists of the following characteristics: income; employment; health and disability; education, skills and training; barriers to housing and services; living environment; and crime. The IMD comes in deciles, ranging from the 1st (least deprived) to the 10th (most deprived). To measure exposure to neighborhood poverty, adolescents who lived in the two most deprived deciles of the IMD were considered to live in a poor neighborhood.

#### Exposure to school poverty

As a commonly used proxy for school poverty (Gorard, 2012), the proportion of children eligible for school meals in the schools that adolescents were attending at the three measurement periods was used (range: 0–100%). This measure is available from the Annual School Census. To measure individual exposure to school poverty, adolescents who attended the poorest 10% of schools were considered to attend a poor school.

#### Duration and timing of exposure to poverty

Both neighborhood and school poverty were studied in two distinct ways. First, the duration of exposure to poverty was defined as the number of periods represented by the three available measurement points (i.e., ages 10/11, 13/14, and 15/16) that adolescents were living in a poor neighborhood or attending a poor school. Both range from 0 to 3. Second, the timing of exposure was operationalized through three dummy variables for the three measurement points. Finally, to measure cumulative poverty in both contexts, the two measures above were merged. First, the duration of exposure was measured by adding the measures for the duration of exposure to neighborhoods and schools (range: 0–6). Second, the timing of the exposure measures for each of the three measurement points and whether adolescents were exposed to poverty in one or two contexts, or whether they were not exposed to poverty (range: 0–2) were determined. To provide insight into the percentage of adolescents who experience poverty at different time points and for multiple time points, the percentages for both contexts are shown in Tables 3 and 4.

**Table 3:** Periods of exposure to poverty (%) (N = 4502)

Context	0	1	2	3	4	5	6
Neighborhood	83.90	2.78	2.02	11.31			
School	85.36	7.29	4.51	2.84			
Both	78.28	5.04	3.71	5.35	3.22	2.22	2.18

**Table 4:** Exposure to poverty at different periods (%) (N = 4502)

Context	No	Yes/One	Both
Neighborhood at age 10/11	86.38	13.62	
Neighborhood at age 13/14	86.58	13.42	
Neighborhood at age 15/16	86.29	13.71	
School at age 10/11	91.23	8.77	
School at age 13/14	92.63	7.37	
School at age 15/16	91.31	8.69	
Both at age 10/11	84.18	9.24	6.57
Both at age 13/14	83.21	12.79	4.00
Both at age 15/16	82.30	13.02	4.69

#### Control variables

Controls for highest level of parental education, gender, previous educational attainment, and moving were included. Level of parental education was measured as the highest education received by either of the parents based on the national standardized tests General Certificate of Secondary Education (GCSE) taken at age 16. For older parents, educational systems preceding GCSEs may apply, specifically the Certificate of Secondary Education (CSE) and Ordinary Level (O Level). Parents’ highest education was divided into five categories, from lowest to highest educated: 1) CSE or GCSE levels D, E, F, or G; 2) vocational; 3) O Level or GCSE levels A, B, or C; 4) Advanced Level (A Level) (an academic preparatory test taken after the GCSEs); and 5) university degree. Gender was included to the models as female = 1 and male = 0. Previous educational attainment was measured as the summary score of the adolescents’ Key Stage 1 test results, a national test administered around age 7 measuring English and math. Moving was measured as whether the adolescents had moved to a different neighborhood between the three measurement periods.

### Analyses

Cross-classified multilevel models were used to study the combined effects of neighborhoods and schools. The structure of the data is such that individuals are nested in neighborhoods and schools, but schools are not nested within neighborhoods. This represents a cross-classified data structure, which can be taken into account with cross-classified multilevel models and thus does not ignore the interdependencies between individuals within both contexts. The proportions of variation in educational attainment due to differences in neighborhoods and schools (i.e., intraclass correlations) were calculated by dividing neighborhood- and school-level variance by the total variance. The intra-neighborhood correlation was 0.088, and the intra-school correlation was 0.133, suggesting that the cross-classified models are justified. To run these models, MlwiN 2.35 through Stata 15.1 using the user written runmlwin command was used (Leckie & Charlton, 2013). MlwiN uses Markov chain Monte Carlo (MCMC) methods to calculate these models. Iterative generalized least squares (IGLS) estimates of the models were used as initial values for the MCMC model parameters.

Three sets of analyses were performed to test the different sets of hypotheses. First, count variables of years of exposure to school poverty and neighborhood poverty were studied to examine the role of duration. The model was first run with neighborhood poverty only and then school poverty was included. Second, to test the timing hypotheses, dummy variables were used to indicate whether respondents had been exposed to poverty in the neighborhood or school context at the three different measurement periods. Again, first a model was assessed with only neighborhood poverty and then with both neighborhood and school poverty. Finally, neighborhood and school poverty were combined in a measure for cumulative contextual poverty. The first model assessed the duration hypothesis, where respondents could be exposed to between zero and six instances of either school or neighborhood poverty. Next, for the timing hypothesis, each measurement period was examined to determine whether respondents were (0) not exposed to poverty, (1) exposed to either school or neighborhood poverty, or (2) exposed to both school and neighborhood poverty. This final set of analyses has the downside that they cannot analyze the differences between neighborhood and school effects, but these were already present in the previous models. The upside is that they allow for a better cumulative measure of experienced poverty than models with separate contexts.

## Results

To gain insight into the combination of adolescents’ exposures to neighborhood and school poverty, both were cross-tabulated to see how many people experience how much poverty in each context separately and then combined (Table 5). The results show that most adolescents were not exposed to poverty in either context. What also stands out is that most adolescents who were exposed to neighborhood poverty were exposed for the maximum of three periods, while exposure to school poverty shows a decreasing number of individuals. Most adolescents who experienced school poverty were exposed for only one period, and the smallest group was those exposed to three periods of school poverty. This suggests that neighborhood poverty is more persistent than school poverty and that neighborhood and school poverty are not perfectly correlated. Adolescents who were exposed to three periods of neighborhood poverty also had higher rates of exposure to school poverty and the highest rate of exposure to three periods of school poverty.

**Table 5:** Individuals in sample with periods of exposure to neighborhood poverty by periods of exposure to school poverty

	Periods of exposure to school poverty				
Periods of exposure to neighborhood poverty	0	1	2	3	Total
0	3524	151	87	15	3777
1	76	26	17	6	125
2	54	20	8	9	91
3	189	131	91	98	509
Total	3843	328	203	128	4502

Model 1 of Table 6 examines the influence of the duration of exposure to neighborhood poverty on educational attainment. One or two periods of exposure to neighborhood poverty show no difference from no exposure to neighborhood poverty. Adolescents who were exposed for three periods had lower levels of educational attainment than adolescents who were not exposed to neighborhood poverty, which is in support of hypothesis 2a that longer exposure to neighborhood poverty is related to lower educational attainment. Model 2 of Table 6 includes the duration of exposure to school poverty. Although the coefficient of being exposed to three periods of neighborhood poverty and the proportion of variance explained by the neighborhood level both decrease, the decrease is not significant thus, there is no support for hypothesis 1 that neighborhood effects are smaller when the school context is taken into account. In line with hypothesis 2b, the duration of exposure to school poverty predicts educational attainment: Adolescents who were exposed for two or three periods of school poverty had lower levels of educational attainment. Furthermore, previous educational attainment and parental education both positively predict educational attainment. Additionally, the more times adolescents moved, the lower their educational attainment.

**Table 6:** Cross-classified multilevel analyses for educational attainment: Duration of exposure to neighborhood and school poverty (N = 4502)

	M1			M2		
	B	SE	p	B	SE	p
Periods of exposure to neighbohood poverty (ref. = 0)						
1	−0.03	0.06	0.632	−0.01	0.06	0.909
2	−0.10	0.07	0.144	−0.07	0.07	0.274
3	−0.21	0.04	<0.001	−0.15	0.04	<0.001
Periods of exposure to school poverty (ref. = 0)						
1				−0.02	0.04	0.579
2				−0.14	0.05	0.009
3				−0.39	0.08	<0.001
Previous educational attainment	0.15	0.00	<0.001	0.15	0.00	<0.001
Parental educ. (ref.=CSE or GCSE (D–G))						
Vocational	0.05	0.04	0.267	0.04	0.04	0.317
O level or GCSE (A–C)	0.18	0.03	<0.001	0.18	0.03	<0.001
A level	0.33	0.03	<0.001	0.33	0.03	<0.001
University degree	0.62	0.04	<0.001	0.62	0.04	<0.001
Gender (female)	−0.03	0.02	0.147	−0.03	0.02	0.124
Times moved (ref. = 0)						
1	−0.06	0.03	0.047	−0.06	0.03	0.035
2	−0.31	0.11	0.005	−0.33	0.11	0.004
Intercept	−10.64	0.04	<0.001	−1.61	0.04	<0.001
Neighborhood-level variance	0.01	0.00		0.01	0.00	
School-level variance	0.02	0.01		0.01	0.00	
Individual-level variance	0.37	0.01		0.37	0.01	
Proportion neighborhood-level variance	0.03			0.02		
Proportion school-level variance	0.04			0.03		
Proportion individual-level variance	0.93			0.95		

Model 1 of Table 7 includes the timing of exposure to neighborhood poverty, but no effects were found for the three different periods of exposure to neighborhood poverty, suggesting that cumulative exposure (see Table 6) is more important when predicting educational attainment. Thus, no support is found for the hypothesis that the timing of exposure to neighborhood poverty is differentially related to educational attainment (hypothesis 3a). Model 2 of Table 7 includes the timing of exposure to school poverty, and somewhat in line with hypothesis 3b, exposure to school poverty at age 13/14 is more strongly related to educational attainment than exposure at both earlier and later ages. Additionally, adolescents who moved in a later period had lower educational attainment than adolescents who moved in an earlier period.

**Table 7:** Cross-classified multilevel analyses for educational attainment: timing of exposure to neighborhood and school poverty (N = 4502)

	M1			M2		
	B	SE	p	B	SE	p
Exposure to neighborhood poverty at age 10/11	−0.03	0.05	0.543	0.01	0.05	0.908
Exposure to neighborhood poverty at age 13/14	−0.05	0.09	0.571	−0.05	0.09	0.582
Exposure to neighborhood poverty at age 15/16	−0.12	0.08	0.147	−0.09	0.08	0.250
Exposure to school poverty at age 10/11				−0.11	0.04	0.006
Exposure to school poverty at age 13/14				−0.21	0.06	0.001
Exposure to school poverty at age 15/16				0.04	0.06	0.496
Previous educational attainment	0.15	0.00	<0.001	0.15	0.00	<0.001
Parental educ. (ref. = CSE or GCSE (D–G))						
Vocational	0.05	0.04	0.263	0.04	0.04	0.301
O level or GCSE (A–C)	0.18	0.03	<0.001	0.18	0.03	<0.001
A level	0.33	0.03	<0.001	0.33	0.03	<0.001
University degree	0.62	0.04	<0.001	0.62	0.04	<0.001
Gender (female)	−0.03	0.02	0.162	−0.03	0.02	0.149
Moved between age 10/11 and 13/14	−0.06	0.03	0.056	−0.06	0.03	0.048
Moved between age 13/14 and 15/16	−0.14	0.06	0.018	−0.15	0.06	0.016
Intercept	−1.64	0.04	<0.001	−1.61	0.04	<0.001
Neighborhood-level variance	0.01	0.00		0.01	0.00	
School-level variance	0.02	0.01		0.01	0.00	
Individual-level variance	0.37	0.01		0.37	0.01	
Proportion neighborhood-level variance	0.03			0.02		
Proportion school-level variance	0.04			0.03		
Proportion individual-level variance	0.93			0.95		

Model 1 of Table 8 includes the duration of exposure to contextual poverty, which includes both exposure to neighborhood poverty and to school poverty. Adolescents who were exposed to contextual poverty once or twice showed no differences in educational attainment from those who were never exposed; however, adolescents who were exposed three times or more had lower levels of educational attainment. The number of exposures is incremental with the effect size, suggesting that increased exposure to multiple contexts of poverty is related to lower levels of educational attainment compared to lower numbers of exposure. Being exposed to six instances of contextual poverty was found to be related to lower educational attainment than being exposed to four instances (b = 0.28, s.e. = 0.09, p = 0.002) or fewer, and being exposed to five instances of poverty was related to lower educational attainment than two exposures (b = 0.33, s.e. = 0.08, p < 0.001) or fewer. This is in line with hypothesis 4, which predicts that longer exposure is related to lower attainment. Model 2 of Table 8 examines the importance of the timing of exposure to contextual poverty. Being exposed to contextual poverty at ages 10/11 and 13/14 was related to educational attainment, albeit only with a significance level of 10% for age 10/11. Exposure at age 15/16 was not related to educational attainment. This finding is somewhat in line with hypothesis 5 that exposure to contextual poverty at ages 13–16 is more strongly related to educational attainment than at earlier ages.

**Table 8:** Cross-classified multilevel analyses for educational attainment: cumulative contextual poverty (N = 4502)

	M1			M2		
	B	SE	p	B	SE	p
Periods of exposure to contextual poverty (ref. = 0)						
1	−0.06	0.05	0.192			
2	0.02	0.05	0.772			
3	−0.18	0.04	<0.001			
4	−0.19	0.06	0.001			
5	−0.32	0.07	<0.001			
6	−0.47	0.08	<0.001			
Cumulative exposure at age 10/11 (ref. = no)						
one context				−0.08	0.04	0.057
two contexts				−0.10	0.06	0.080
Cumulative exposure at age 13/14 (ref. = no)						
one context				−0.05	0.06	0.384
two contexts				−0.34	0.11	0.001
Cumulative exposure at age 15/16 (ref. = no)						
one context				−0.02	0.06	0.728
two contexts				−0.02	0.10	0.841
Previous educational attainment	0.15	0.00	<0.001	0.15	0.00	<0.001
Parental educ. (ref. = CSE or GCSE (D–G))						
Vocational	0.04	0.04	0.302	0.04	0.04	0.287
O level or GCSE (A–C)	0.18	0.03	<0.001	0.18	0.03	<0.001
A level	0.33	0.03	<0.001	0.33	0.03	<0.001
University degree	0.62	0.04	<0.001	0.62	0.04	<0.001
Gender (female)	−0.03	0.02	0.132	−0.03	0.02	0.142
Times moved (ref. = 0)						
1	−0.06	0.03	0.036			
2	−0.32	0.11	0.004			
Moved between age 10/11 and 13/14				−0.05	0.03	0.068
Moved between age 13/14 and 15/16				−0.14	0.06	0.016
Intercept	−1.62	0.04	<0.001	−1.61	0.04	<0.001
Neighborhood-level variance	0.01	0.00		0.01	0.00	
School-level variance	0.01	0.00		0.01	0.00	
Individual-level variance	0.37	0.01		0.37	0.01	
Proportion neighborhood-level variance	0.03			0.02		
Proportion school-level variance	0.03			0.03		
Proportion individual-level variance	0.94			0.95		

To address potential selection bias arising from children with a certain educational advantage going to different schools or living in different neighborhoods than children with a certain educational disadvantage, a pretreatment control variable was included in the models, which is previous educational attainment, as measured approximately three years before the first measurement period for neighborhood and school poverty. This (partly) takes care of the variance potentially introduced by hereditary factors or the different learning environments provided by parents from different socioeconomic backgrounds. All models were run without previous educational attainment as well (see Appendix A), and the results indeed indicate selection bias, as these models have larger and more often significant coefficients.

Two extra sets of sensitivity analyses were run. First, because a factor score of three test results (i.e., reading, math, and science) was used as the outcome variable, all models were also assessed with the results of the three different tests separately (see Appendix B). The results of these sensitivity analyses lead to exactly the same interpretation as the results from the factor score for attainment. Therefore, for brevity, only the results of the factor score are presented. Second, a different set of cutoff points for neighborhood and school poverty was used to demonstrate the robustness of the measures presented in this study (see Appendix C). The only change in interpretation is that the late timing of exposure to school poverty also seems to play a role. The overall robustness of the cutoff points is adequate.

## Discussion

Many studies on the role of contextual characteristics in educational attainment often restrict themselves to one context, such as neighborhood or school environments, at one point in time. To address this gap, this study specifically studies both contexts at the same time over a period of six years. Studying the combined effects of neighborhood poverty and school poverty on adolescents’ educational attainment is of crucial importance when trying to understand how context relates to adolescents’ chances in life. Therefore, this research studied how educational attainment at age 16 in England is affected by simultaneous exposure to three periods of neighborhood poverty and school poverty. By examining three different periods of exposure to poverty, differential effects of early vs. late exposure and prolonged exposure vs. short spells of exposure to contextual poverty were also disentangled. This resulted in a study of the role of timing and duration of exposure to neighborhood and school poverty in adolescents’ educational attainment.

The effect of exposure to neighborhood poverty was expected to be diminished by including school poverty (hypothesis 1); however, no support for this expectation was found. This suggests that neighborhood poverty impacts adolescents’ educational attainment independent of school poverty. This is in line with a recent study that did not find a mediating effect of school poverty on the relation between neighborhood poverty and educational attainment in the US (Wodtke & Parbst, 2017); however, it is not in line with a study from the Netherlands that found that neighborhood effects disappear after including school characteristics (Sykes & Musterd, 2011) or studies where the neighborhood effect partly disappears, as in Finland (Brännström, 2008; Kauppinen, 2008). It is not unlikely that the much steeper levels of segregation in the UK and the US also result in a higher likelihood of finding neighborhood effects, whereas in countries such as the Netherlands and Finland, with much lower levels of segregation and fewer neighborhoods with high poverty concentrations, neighborhoods are of less importance when predicting educational attainment (Nieuwenhuis & Hooimeijer, 2016; Nieuwenhuis et al., 2020). The lack of support for hypothesis 1 therefore may be due to the UK inequality levels being more comparable to those of the US than to those of the Netherlands or Finland (Roser & Ortiz-Ospina, 2013). In the UK, neighborhood effects are not explained through school effects, but neighborhood poverty leads to lower educational attainment, despite the school adolescents attend. Perhaps because children from different socioeconomic groups from the same neighborhood potentially attend different schools (Burgess et al., 2011; Nieuwenhuis & Xu, 2021), the socioeconomic composition of both contexts might not actually overlap that much. However, because the sample was quite specific, in that there was an underrepresentation of migrants and lower-class individuals, it is difficult to say how far this result can be generalized. Future studies should target a broader population, which could result in higher variability in terms of neighborhood and school poverty and therefore potentially different results.

Next, duration and timing effects were examined. First, in line with hypotheses 2a and 2b, longer exposure to either neighborhood (a) or school poverty (b) was found to relate to lower educational attainment. Adolescents who were exposed for only one period to school poverty or one or two periods to neighborhood poverty did not have different educational attainment compared to unexposed adolescents. However, exposure to two or three periods of school poverty or three periods of neighborhood poverty led to lower educational attainment. This corroborates other studies (Hicks et al., 2018; Wodtke et al., 2011) that find that prolonged exposure has a more severe impact on adolescents’ educational outcomes than short-term exposure. This finding again highlights the importance of studying enduring exposure to contextual poverty, rather than the more common cross-sectional approach of studying exposure and outcomes at the same point in time.

Second, the effect of the timing of exposure to poverty was studied. The hypothesis was that exposure to neighborhood (hypothesis 3a) and school poverty (hypothesis 3b) at ages 13–16 is more strongly related to educational attainment than such exposures at earlier ages (10/11). For neighborhood poverty, no differences were found in adolescents’ educational attainment depending on whether they were exposed at an early age or a later age, thus offering no support for hypothesis 3a. When not taking into account prior educational attainment, exposure to neighborhood poverty at a later age was found to have a stronger effect than it had at an earlier age. However, this finding seems to be driven by selection only. Furthermore, somewhat in line with hypothesis 3b, exposure to school poverty at age 13/14 is more strongly related to educational attainment than exposure at age 10/11 or at 15/16. For neighborhood poverty, this suggests that enduring exposure is especially important, but within the timeframe of the study, the timing of this exposure does not play an important role. However, for school poverty, both timing and duration seem to play a role when predicting educational attainment. Why this difference exists between neighborhoods and schools is unclear. Earlier exposure has a stronger impact on educational attainment than later exposure. It has been argued that interventions in early-age contextual poverty are more beneficial for later life income than interventions during ages late in adolescence (Chetty et al., 2015). Other studies have suggested that during age 13/14 adolescents are the most susceptible to peer pressure and to the norms favored by their peers (Steinberg, 2008; Zohar et al., 2019). The finding that exposure to school poverty at age 13/14 has a stronger impact on educational attainment than exposure at other ages supports the finding that interventions with young people are most beneficial when targeting issues of educational stratification in a school context. However, the results also nuance this finding by pointing out that at different ages, interventions will have a different impact. Future studies could elaborate on this by taking into account much more detailed information on timing, rather than only three measurements.

Finally, the cumulative exposure to either neighborhood and/or school poverty was studied. In line with the separate hypotheses for neighborhood and school, here, it was found that the longer the exposure to several contexts of poverty, the lower adolescents’ educational attainment (hypothesis 4), and exposure at age 13/14 was found to be more strongly related to educational attainment than exposure at other ages (hypothesis 5). Combining the contextual measures of poverty showed that a cumulative perspective on contextual poverty adds an additional explanatory perspective to the study of educational attainment and poverty, which is in line with previous studies of multiple contexts (Whipple et al., 2010). Exposure to one or two periods of contextual poverty does not affect educational attainment; however, the more exposure to neighborhood and school poverty there is, the lower adolescents’ educational attainment. The strongest impact was observed for adolescents who were exposed to poverty for the maximum of three periods in both the neighborhood and the school context.

Parental socioeconomic status normally strongly influences the type of neighborhoods in which adolescents grow up and the quality of the schools they attend. Not properly taking this selection into account can lead to spurious contextual effects that could be explained by the sorting of families with different socioeconomic status into different neighborhoods and schools (Galster, 2008). The first attempt to overcome this used a cross-classified multilevel model that takes into account the multiple membership structure of adolescents in neighborhoods and schools. This provides the better estimation of standard errors of the higher level predictor variables, thereby not overestimating the significance of contextual predictors. Second, parental education and adolescents’ own previously attained educational results were controlled for. Parental education partly deals with the potential bias that can arise from parental selection into neighborhoods based on their educational background and the schools they choose for their children. Adolescents’ previous educational attainment was measured pretreatment, that is, just before the periods of exposure to contextual poverty that were studied. This partly controls for confounding introduced through the parents. For example, selection due to heritability of educational attainment (Okbay et al., 2016) is partly captured by this measure: If parents who have a higher education also have more highly educated children as well as better paying jobs that can buy them into wealthier neighborhoods and better schools, this could introduce a bias. The same argument applies to more highly educated parents being able to provide their children with the appropriate cultural capital to succeed in education (Bourdieu & Passeron, 1990), as well as having jobs with better salaries that allow them access to low-poverty contexts. By controlling for educational attainment just before the study period, much of this variance was removed. The models that did not include prior educational attainment indeed showed stronger neighborhood and school effects. This suggests that the models that take prior educational attainment into account address some of the selection bias and are more indicative of the influence of neighborhoods and schools on educational attainment.

Although the absolute measures of neighborhood and school poverty provided a good picture of the contextual disadvantages faced by the adolescents in the sample, they fail to show adolescents’ relative standing within those contexts. Even though parental education is taken into account to control for adolescents’ socioeconomic background, a clear measure of adolescents’ relative socioeconomic position within their neighborhood and school is missing. Previous studies have shown that relative deprivation in neighborhood and school contexts contributes to adolescents’ outcomes (Nieuwenhuis et al., 2017; Owens, 2010). Future studies should obtain measures that pertain to both absolute and relative contextual poverty to arrive at a more comprehensive understanding of the timing and duration of exposure to poverty.

## Conclusion

It is important to examine both neighborhood and school effects simultaneously when studying educational attainment. When either one of the two contexts is studied alone, there is a risk of overestimating the role of that context. Neighborhood contexts and school contexts can be similar but are not the same in demographic composition. Studying both in a multiple membership framework allows for the disentanglement of the contexts, resulting in better estimates. For adolescents, when growing up, neighborhoods and schools are two salient environments where they spend much time. Studying them both in a framework that takes into account how much time is spent in the poorest contexts helps to clarify the importance of contextual poverty for educational attainment. Since prolonged and early exposure to contextual poverty seems most harmful, when interventions are considered, it is important to implement them early in adolescents’ lives.

## Appendix A

This appendix shows the results of Tables 6 through 8 without including previous educational attainment (Tables 9 through 11). The coefficients of exposure to neighborhood and school poverty in the tables below are larger and more often significant compared to the coefficients in Tables 6 through 8, which include previous educational attainment. This indicates that neighborhood and school effects are partly explained by selection.

Tables 9–11

**Table 9:** Cross-classified multilevel analyses for educational attainment: Duration of exposure to neighborhood and school poverty (N = 4502)

	M1			M2		
	B	SE	p	B	SE	p
Periods of exposure to neighborhood poverty (ref. = 0)						
1	−0.14	0.08	0.061	−0.11	0.08	0.161
2	−0.26	0.09	0.002	−0.22	0.09	0.012
3	−0.38	0.04	<0.001	−0.29	0.05	<0.001
Periods of exposure to school poverty (ref. = 0)						
1				−0.07	0.05	0.208
2				−0.25	0.07	<0.001
3				−0.54	0.10	<0.001
Parental educ. (ref. = CSE or GCSE (D–G))						
Vocational	0.06	0.05	0.237	0.06	0.05	0.251
O level or GCSE (A–C)	0.40	0.04	<0.001	0.39	0.04	<0.001
A level	0.60	0.04	<0.001	0.60	0.04	<0.001
University degree	1.12	0.05	<0.001	1.12	0.05	<0.001
Gender (female)	0.12	0.02	<0.001	0.12	0.02	<0.001
Times moved (ref. = 0)						
1	−0.06	0.04	0.088	−0.07	0.04	0.069
2	−0.23	0.14	0.098	−0.26	0.14	0.070
Intercept	−0.50	0.05	<0.001	−0.45	0.05	<0.001
Neighborhood-level variance	0.02	0.01		0.02	0.01	
School-level variance	0.03	0.01		0.02	0.01	
Individual-level variance	0.60	0.01		0.60	0.01	
Proportion neighborhood-level variance	0.03			0.03		
Proportion school-level variance	0.05			0.03		
Proportion individual-level variance	0.93			0.94

**Table 10:** Cross-classified multilevel analyses for educational attainment: timing of exposure to neighborhood and school poverty (N = 4502)

	M1			M2		
	B	SE	p	B	SE	p
Exposure to neighborhood poverty at age 10/11	−0.07	0.07	0.280	−0.02	0.07	0.771
Exposure to neighborhood poverty at age 13/14	−0.05	0.11	0.665	−0.04	0.11	0.742
Exposure to neighborhood poverty at age 15/16	−0.26	0.10	0.012	−0.22	0.10	0.029
Exposure to school poverty at age 10/11				−0.17	0.05	0.002
Exposure to school poverty at age 13/14				−0.22	0.08	0.007
Exposure to school poverty at age 15/16				−0.05	0.08	0.507
Parental educ. (ref. = CSE or GCSE (D–G))						
Vocational	0.06	0.05	0.239	0.06	0.05	0.246
O level or GCSE (A–C)	0.40	0.04	<0.001	0.39	0.04	<0.001
A level	0.60	0.04	<0.001	0.60	0.04	<0.001
University degree	1.12	0.05	<0.001	1.12	0.05	<0.001
Gender (female)	0.12	0.02	<0.001	0.12	0.03	<0.001
Moved between age 10/11 and 13/14	−0.08	0.04	0.031	−0.09	0.04	0.024
Moved between age 13/14 and 15/16	−0.07	0.08	0.351	−0.08	0.08	0.306
Intercept	−0.50	0.05	<0.001	−0.45	0.05	<0.001
Neighborhood-level variance	0.02	0.01		0.02	0.01	
School-level variance	0.03	0.01		0.02	0.01	
Individual-level variance	0.60	0.01		0.60	0.01	
Proportion neighborhood-level variance	0.03			0.03		
Proportion school-level variance	0.05			0.03		
Proportion individual-level variance	0.93			0.94

**Table 11:** Cross-classified multilevel analyses for educational attainment: cumulative contextual poverty (N = 4502)

	M1			M2		
	B	SE	p	B	SE	p
Periods of exposure to contextual poverty (ref. = 0)						
1	−0.13	0.06	0.037			
2	−0.08	0.07	0.242			
3	−0.31	0.05	<0.001			
4	−0.42	0.07	<0.001			
5	−0.58	0.09	<0.001			
6	−0.74	0.10	<0.001			
Cumulative exposure at age 10/11 (ref. = no)						
one context				−0.08	0.06	0.166
two contexts				−0.19	0.07	0.007
Cumulative exposure at age 13/14 (ref. = no)						
one context				−0.07	0.07	0.363
two contexts				−0.35	0.13	0.006
Cumulative exposure at age 15/16 (ref. = no)						
one context				−0.12	0.07	0.092
two contexts				−0.23	0.13	0.073
Parental educ. (ref. = CSE or GCSE (D–G))						
Vocational	0.06	0.05	0.250	0.06	0.05	0.252
O level or GCSE (A–C)	0.39	0.04	<0.001	0.39	0.04	<0.001
A level	0.60	0.04	<0.001	0.60	0.04	<0.001
University degree	1.11	0.05	<0.001	1.12	0.05	<0.001
Gender (female)	0.12	0.02	<0.001	0.12	0.02	<0.001
Times moved (ref. = 0)						
1	−0.07	0.04	0.049			
2	−0.26	0.14	0.066			
Moved between age 10/11 and 13/14				−0.08	0.04	0.031
Moved between age 13/14 and 15/16				−0.08	0.08	0.300
Intercept	−0.46	0.05	<0.001	−0.46	0.05	<0.001
Neighborhood-level variance	0.02	0.01		0.02	0.01	
School-level variance	0.02	0.01		0.02	0.01	
Individual-level variance	0.60	0.01		0.60	0.01	
Proportion neighborhood-level variance	0.03			0.03		
Proportion school-level variance	0.03			0.03		
Proportion individual-level variance	0.94			0.94		

## Appendix B

Appendix B shows the descriptive statistics (Table 12) and the results from Tables 6 through 8 for the separate Key Stage 4 test results for the subjects reading (Tables 13–15), math (Tables 16–18), and science Tables 19–21).

Tables 12–21

**Table 12:** Descriptive statistics (N = 4502)

Variable	Mean	SD	Min.	Max.
Reading	390.49	10.16	0	58
Math	38.33	11.32	0	68
Science	40.86	9.94	0	58

**Table 13:** Cross-classified multilevel analyses for educational attainment (reading): Duration of exposure to neighborhood and school poverty (N = 4502)

	M1			M2		
	B	SE	p	B	SE	p
Periods of exposure to neighborhood poverty (ref. = 0)						
1	−0.63	0.72	0.383	−0.46	0.73	0.524
2	−1.32	0.85	0.121	−1.04	0.83	0.211
3	−1.79	0.44	<0.001	−1.28	0.47	0.006
Periods of exposure to school poverty (ref. = 0)						
1				−0.82	0.50	0.105
2				−0.07	0.67	0.923
3				−3.23	0.92	<0.001
Previous educational attainment	1.46	0.03	<0.001	1.45	0.04	<0.001
Parental educ. (ref. = CSE or GCSE (D–G))						
Vocational	1.20	0.52	0.020	1.14	0.52	0.028
O level or GCSE (A–C)	2.31	0.37	<0.001	2.26	0.37	<0.001
A level	3.59	0.37	<0.001	3.56	0.38	<0.001
University degree	6.46	0.46	<0.001	6.42	0.47	<0.001
Gender (female)	2.59	0.23	<0.001	2.56	0.23	<0.001
Times moved (ref. = 0)						
1	−0.89	0.36	0.012	−0.91	0.35	0.010
2	−4.05	1.38	0.003	−4.12	1.38	0.003
Intercept	21.49	0.50	<0.001	21.75	0.50	<0.001
Neighborhood-level variance	1.66	0.50		1.56	0.59	
School-level variance	1.78	0.97		1.33	0.67	
Individual-level variance	56.38	1.26		56.39	1.29	
Proportion neighborhood-level variance	0.03			0.03		
Proportion school-level variance	0.03			0.02		
Proportion individual-level variance	0.94			0.95

**Table 14:** Cross-classified multilevel analyses for educational attainment (reading): timing of exposure to neighborhood and school poverty (N = 4502)

	M1			M2		
	B	SE	p	B	SE	p
Exposure to neighborhood poverty at age 10/11	−0.31	0.64	0.627	−0.12	0.65	0.852
Exposure to neighborhood poverty at age 13/14	−1.08	1.09	0.322	−1.09	1.08	0.316
Exposure to neighborhood poverty at age 15/16	−0.39	1.00	0.696	−0.19	1.00	0.850
Exposure to school poverty at age 10/11				−1.47	0.51	0.004
Exposure to school poverty at age 13/14				−2.09	0.76	0.006
Exposure to school poverty at age 15/16				1.50	0.78	0.053
Previous educational attainment	1.46	0.03	<0.001	1.45	0.04	<0.001
Parental educ. (ref. = CSE or GCSE (D–G))						
Vocational	1.20	0.52	0.020	1.16	0.52	0.025
O level or GCSE (A–C)	2.31	0.37	<0.001	2.25	0.37	<0.001
A level	2.59	0.37	<0.001	3.55	0.38	<0.001
University degree	6.46	0.46	<0.001	6.42	0.47	<0.001
Gender (female)	2.59	0.23	<0.001	2.58	0.23	<0.001
Moved between age 10/11 and 13/14	−1.05	0.37	0.004	−1.06	0.36	0.004
Moved between age 13/14 and 15/16	−1.43	0.74	0.053	−1.43	0.74	0.054
Intercept	21.49	0.50	<0.001	21.68	0.51	<0.001
Neighborhood-level variance	1.65	0.50		1.53	0.59	
School-level variance	1.81	0.98		1.55	0.82	
Individual-level variance	56.40	1.26		56.40	1.29	
Proportion neighborhood-level variance	0.03			0.03		
Proportion school-level variance	0.03			0.03		
Proportion individual-level variance	0.94			0.95

**Table 15:** Cross-classified multilevel analyses for educational attainment (reading): cumulative contextual poverty (N = 4502)

	M1			M2		
	B	SE	p	B	SE	p
Periods of exposure to contextual poverty (ref. = 0)						
1	−1.13	0.57	0.048			
2	0.99	0.64	0.122			
3	−1.54	0.55	0.005			
4	−2.46	0.69	<0.001			
5	−1.88	0.88	0.032			
6	−4.15	0.94	<0.001			
Cumulative exposure at age 10/11 (ref. = no)						
one context				−0.92	0.52	0.081
two contexts				−1.70	0.68	0.013
Cumulative exposure at age 13/14 (ref. = no)						
one context				−0.45	0.76	0.553
two contexts				−4.15	1.28	0.001
Cumulative exposure at age 15/16 (ref. = no)						
one context				−0.18	0.73	0.802
two contexts				−1.88	1.25	0.133
Previous educational attainment	1.45	0.04	<0.001	1.46	0.04	<0.001
Parental educ. (ref. = CSE or GCSE (D–G))						
Vocational	1.15	0.52	0.026	1.18	0.52	0.023
O level or GCSE (A–C)	2.25	0.37	<0.001	2.26	0.37	<0.001
A level	3.54	0.38	<0.001	3.56	0.38	<0.001
University degree	6.41	0.47	<0.001	6.44	0.47	<0.001
Gender (female)	2.57	0.23	<0.000	2.57	0.23	<0.000
Times moved (ref. = 0)						
1	−0.96	0.35	0.006			
2	−40.21	1.38	0.002			
Moved between age 10/11 and 13/14				−1.00	0.36	0.006
Moved between age 13/14 and 15/16				−1.42	0.74	0.056
Intercept	21.73	0.50	<0.001	21.68	0.50	<0.001
Neighborhood-level variance	1.58	0.59		1.57	0.59	
School-level variance	1.35	0.69		1.39	0.70	
Individual-level variance	56.29	1.29		56.35	1.29	
Proportion neighborhood-level variance	0.03			0.03		
Proportion school-level variance	0.02			0.02		
Proportion individual-level variance	0.95			0.95

**Table 16:** Cross-classified multilevel analyses for educational attainment (math): Duration of exposure to neighborhood and school poverty (N = 4502)

	M1			M2		
	B	SE	p	B	SE	p
Periods of exposure to neighborhood poverty (ref. = 0)						
1	−0.03	0.78	0.971	−0.31	0.78	0.691
2	−1.54	0.91	0.092	−1.25	0.89	0.163
3	−2.32	0.46	<0.001	−1.63	0.50	0.001
Periods of exposure to school poverty (ref. = 0)						
1				−0.05	0.54	0.930
2				−2.81	0.73	<0.001
3				−4.49	1.01	<0.001
Previous educational attainment	1.82	0.04	<0.001	1.81	0.04	<0.001
Parental educ. (ref. = CSE or GCSE (D–G))						
Vocational	0.70	0.56	0.210	0.67	0.56	0.232
O level or GCSE (A–C)	2.13	0.40	<0.001	2.08	0.40	<0.001
A level	3.92	0.40	<0.001	3.91	0.41	<0.001
University degree	7.01	0.50	<0.001	7.03	0.50	<0.001
Gender (female)	−1.32	0.25	<.001	−1.32	0.25	<0.001
Times moved (ref. = 0)						
1	−0.39	0.38	0.309	-0.42	0.38	0.267
2	−4.29	1.49	0.004	−4.45	1.50	0.003
Intercept	19.05	0.56	<0.001	19.39	0.55	<0.001
Neighborhood-level variance	1.21	0.52		1.02	0.55	
School-level variance	3.09	1.24		1.91	0.76	
Individual-level variance	66.38	1.48		66.39	1.48	
Proportion neighborhood-level variance	0.02			0.01		
Proportion school-level variance	0.04			0.03		
Proportion individual-level variance	0.94			0.96

**Table 17:** Cross-classified multilevel analyses for educational attainment (math): timing of exposure to neighborhood and school poverty (N = 4502)

	M1			M2		
	B	SE	p	B	SE	p
Exposure to neighborhood poverty at age 10/11	−0.09	0.69	0.902	0.35	0.70	0.613
Exposure to neighborhood poverty at age 13/14	−1.86	1.18	0.114	−1.80	1.17	0.125
Exposure to neighborhood poverty at age 15/16	−0.30	1.07	0.778	−0.02	1.07	0.987
Exposure to school poverty at age 10/11				−1.32	0.55	0.016
Exposure to school poverty at age 13/14				−1.61	0.82	0.050
Exposure to school poverty at age 15/16				−0.80	0.82	0.330
Previous educational attainment	1.82	0.04	<0.001	1.81	0.04	<0.001
Parental educ. (ref. = CSE or GCSE (D–G))						
Vocational	0.70	0.56	0.208	0.67	0.56	0.234
O level or GCSE (A–C)	2.14	0.40	<0.001	2.08	0.40	<0.001
A level	3.90	0.40	<0.001	3.87	0.41	<0.001
University degree	7.00	0.50	<0.001	6.97	0.50	<0.001
Gender (female)	−1.31	0.25	<0.001	−1.32	0.25	<0.001
Moved between age 10/11 and 13/14	−0.49	0.40	0.215	−0.52	0.40	0.191
Moved between age 13/14 and 15/16	−1.45	0.80	0.071	−1.49	0.80	0.063
Intercept	19.07	0.57	<0.001	19.46	0.56	<0.001
Neighborhood-level variance	1.25	0.51		1.05	0.55	
School-level variance	3.09	1.24		2.05	0.82	
Individual-level variance	66.40	1.48		66.49	1.48	
Proportion neighborhood-level variance	0.02			0.02		
Proportion school-level variance	0.04			0.03		
Proportion individual-level variance	0.94			0.96

**Table 18:** Cross-classified multilevel analyses for educational attainment (math): cumulative contextual poverty (N = 4502)

	M1			M2		
	B	SE	p	B	SE	p
Periods of exposure to contextual poverty (ref. = 0)						
1	−0.68	0.61	0.266			
2	−0.49	0.69	0.482			
3	−2.29	0.58	<0.001			
4	−1.89	0.74	0.010			
5	−4.76	0.94	<0.001			
6	−4.88	1.04	<0.001			
Cumulative exposure at age 10/11 (ref. = no)						
one context				−0.87	0.57	0.126
two contexts				−0.81	0.74	0.270
Cumulative exposure at age 13/14 (ref. = no)						
one context				−1.13	0.82	0.168
two contexts				−2.78	1.41	0.048
Cumulative exposure at age 15/16 (ref. = no)						
one context				−0.11	0.79	0.892
two contexts				−1.40	1.37	0.307
Previous educational attainment	1.81	0.04	<0.001	1.81	0.04	<0.001
Parental educ. (ref. = CSE or GCSE (D–G))						
Vocational	0.69	0.56	0.218	0.67	0.56	0.234
O level or GCSE (A–C)	2.09	0.40	<0.001	2.08	0.40	<0.001
A level	3.89	0.41	<0.001	3.86	0.41	<0.001
University degree	6.98	0.50	<0.001	6.97	0.50	<0.001
Gender (female)	−1.32	0.25	<0.001	−1.31	0.25	<0.001
Times moved (ref. = 0)						
1	−0.37	0.38	0.324			
2	−4.31	1.50	0.004			
Moved between age 10/11 and 13/14						
Moved between age 13/14 and 15/16						
Intercept	19.36	0.56	<0.001	19.40	0.56	<0.001
Neighborhood-level variance	1.10	0.55		1.06	0.55	
School-level variance	2.12	0.86		2.10	0.84	
Individual-level variance	66.38	1.48		66.50	1.48	
Proportion neighborhood-level variance	0.02			0.02		
Proportion school-level variance	0.03			0.03		
Proportion individual-level variance	0.95			0.95

**Table 19:** Cross-classified multilevel analyses for educational attainment (science): Duration of exposure to neighborhood and school poverty (N = 4502)

	M1			M2		
	B	SE	p	B	SE	p
Periods of exposure to neighborhood poverty (ref. = 0)						
1	−0.39	0.69	0.576	−0.16	0.69	0.813
2	−0.58	0.81	0.471	−0.30	0.79	0.703
3	−2.34	0.41	<0.001	−1.69	0.44	<0.001
Periods of exposure to school poverty (ref. = 0)						
1				−0.11	0.49	0.817
2				−1.49	0.67	0.027
3				−4.68	0.95	<0.001
Previous educational attainment	1.54	0.03	<0.001	1.54	0.03	<0.001
Parental educ. (ref. = CSE or GCSE (D–G))						
Vocational	0.06	0.49	0.908	−0.12	0.49	0.807
O level or GCSE (A–C)	1.54	0.35	<0.001	1.48	0.35	<0.001
A level	3.19	0.35	<0.001	3.16	0.36	<0.001
University degree	6.51	0.44	<0.001	6.49	0.44	<0.001
Gender (female)	−1.07	0.22	<0.001	−1.10	0.22	<0.001
Times moved (ref. = 0)						
1	−0.63	0.34	0.062	−0.67	0.34	0.046
2	−2.23	1.31	0.089	−2.35	1.32	0.075
Intercept	24.65	0.50	<0.001	25.01	0.52	<0.001
Neighborhood-level variance	1.57	0.47		1.43	0.51	
School-level variance	2.72	1.03		2.59	0.94	
Individual-level variance	50.89	1.14		50.77	1.15	
Proportion neighborhood-level variance	0.03			0.03		
Proportion school-level variance	0.05			0.05		
Proportion individual-level variance	0.92			0.93

**Table 20:** Cross-classified multilevel analyses for educational attainment (science): timing of exposure to neighborhood and school poverty (N = 4502)

	M1			M2		
	B	SE	p	B	SE	p
Exposure to neighborhood poverty at age 10/11	−0.56	0.61	0.360	−0.20	0.62	0.741
Exposure to neighborhood poverty at age 13/14	0.82	1.04	0.430	0.80	1.03	0.436
Exposure to neighborhood poverty at age 15/16	−2.49	0.95	0.009	−2.21	0.94	0.019
Exposure to school poverty at age 10/11				−0.94	0.49	0.053
Exposure to school poverty at age 13/14				−3.03	0.75	<0.001
Exposure to school poverty at age 15/16				0.85	0.75	0.259
Previous educational attainment	1.54	0.03	<0.001	1.54	0.03	<0.001
Parental educ. (ref. = CSE or GCSE (D–G))						
Vocational	−0.05	0.49	0.921	−0.09	0.49	0.857
O level or GCSE (A–C)	1.54	0.35	<0.001	1.50	0.35	<0.001
A level	3.17	0.35	<0.001	3.16	0.36	<0.001
University degree	6.50	0.44	<0.001	6.49	0.44	<0.001
Gender (female)	−1.06	0.22	<0.001	−1.07	0.22	<0.001
Moved between age 10/11 and 13/14	−0.45	0.35	0.195	−0.47	0.35	0.173
Moved between age 13/14 and 15/16	−1.62	0.70	0.021	−1.65	0.70	0.019
Intercept	24.67	0.50	<0.001	24.98	0.51	<0.001
Neighborhood-level variance	1.60	0.47		1.42	0.51	
School-level variance	2.71	1.02		2.46	0.89	
Individual-level variance	50.82	1.14		50.77	1.15	
Proportion neighborhood-level variance	0.03			0.03		
Proportion school-level variance	0.05			0.05		
Proportion individual-level variance	0.92			0.93

**Table 21:** Cross-classified multilevel analyses for educational attainment (science): cumulative contextual poverty (N = 4502)

	M1			M2		
	B	SE	p	B	SE	p
Periods of exposure to contextual poverty (ref. = 0)						
1	−0.36	0.55	0.512			
2	0.16	0.61	0.793			
3	−1.95	0.52	<0.001			
4	−1.97	0.66	0.003			
5	−3.14	0.84	<0.001			
6	−5.78	0.92	<0.001			
Cumulative exposure at age 10/11 (ref. = no)						
one context				−0.79	0.50	0.117
two contexts				−0.79	0.65	0.224
Cumulative exposure at age 13/14 (ref. = no)						
one context				−0.21	0.73	0.772
two contexts				−4.21	1.27	0.001
Cumulative exposure at age 15/16 (ref. = no)						
one context				−0.60	0.71	0.393
two contexts				−0.53	1.23	0.664
Previous educational attainment	1.54	0.03	<0.001	1.54	0.03	<0.001
Parental educ. (ref. = CSE or GCSE (D–G))						
Vocational	−0.11	0.49	0.825	−0.07	0.49	0.881
O level or GCSE (A–C)	1.49	0.35	<0.001	1.48	0.35	<0.001
A level	3.15	0.36	<0.001	3.14	0.36	<0.001
University degree	6.48	0.44	<0.001	6.49	0.44	<0.001
Gender (female)	−1.09	0.22	<0.001	−1.08	0.22	<0.001
Times moved (ref. = 0)						
1	−0.65	0.33	0.048			
2	−2.29	1.31	0.081			
Moved between age 10/11 and 13/14				−0.42	0.35	.223
Moved between age 13/14 and 15/16				−1.65	0.70	0.019
Intercept	24.93	0.51	<0.001	24.98	0.51	<0.001
Neighborhood-level variance	1.46	0.51		1.38	0.50	
School-level variance	2.45	0.89		2.53	0.91	
Individual-level variance	50.77	1.15		50.76	1.15	
Proportion neighborhood-level variance	0.03			0.03		
Proportion school-level variance	0.04			0.05		
Proportion individual-level variance	0.93			0.93		

## Appendix C

This appendix replicates the results of Model 2 from Tables 6 and 7 (Tables 22 and 23, respectively). The models present the results, but with different cut-off points for what is defined as a poor neighborhood or school. The original analyses use the two poorest IMD deciles to define neighborhood poverty, this appendix uses a measure that takes the three poorest IMD deciles. The original analyses use the poorest decile based on school meal eligibility to define school poverty, this appendix uses a measure that takes the poorest quarter.

Tables 22, 23

**Table 22:** Cross-classified multilevel analyses for educational attainment: Duration of exposure to neighborhood and school poverty (N = 4502)

	M2		
	B	SE	p
Periods of exposure to neighbohood poverty (ref. = 0)			
1	−0.01	0.05	0.915
2	−0.05	0.06	0.372
3	−0.13	0.03	<0.001
Periods of exposure to school poverty (ref. = 0)			
1	−0.10	0.03	0.003
2	−0.11	0.04	0.008
3	−0.18	0.05	<0.001
Previous educational attainment	0.15	0.00	<0.001
Parental educ. (ref. = CSE or GCSE (D–G))			
Vocational	0.05	0.04	0.269
O level or GCSE (A–C)	0.18	0.03	<0.001
A level	0.33	0.03	<0.001
University degree	0.61	0.04	<0.001
Gender (female)	−0.02	0.02	0.226
Times moved (ref. = 0)			
1	−0.06	0.03	0.040
2	−0.31	0.11	0.006
Intercept	−1.57	0.04	<0.001
Neighborhood-level variance	0.01	0.00	
School-level variance	0.01	0.00	
Individual-level variance	0.37	0.01	
Proportion neighborhood-level variance	0.03		
Proportion school-level variance	0.03		
Proportion individual-level variance	0.94

**Table 23:** Cross-classified multilevel analyses for educational attainment: timing of exposure to neighborhood and school poverty (N = 4502)

	M2		
	B	SE	p
Exposure to neighborhood poverty at age 10/11	0.02	0.05	0.738
Exposure to neighborhood poverty at age 13/14	−0.10	0.08	0.207
Exposure to neighborhood poverty at age 15/16	−0.04	0.07	0.558
Exposure to school poverty at age 10/11	−0.08	0.03	0.006
Exposure to school poverty at age 13/14	0.06	0.05	0.223
Exposure to school poverty at age 15/16	−0.17	0.05	0.001
Previous educational attainment	0.15	0.00	<0.001
Parental educ. (ref. = CSE or GCSE (D–G))			
Vocational	0.05	0.04	0.268
O level or GCSE (A–C)	0.18	0.03	<0.001
A level	0.33	0.03	<0.001
University degree	0.61	0.04	<0.001
Gender (female)	−0.02	0.02	0.226
Moved between age 10/11 and 13/14	−0.05	0.03	0.053
Moved between age 13/14 and 15/16	−0.30	0.11	0.007
Intercept	−1.57	0.04	<0.001
Neighborhood-level variance	0.01	0.00	
School-level variance	0.01	0.01	
Individual-level variance	0.37	0.01	
Proportion neighborhood-level variance	0.03		
Proportion school-level variance	0.03		
Proportion individual-level variance	0.94		
